# Evaluation of the hoof’s temperature variations depending on lesion presence, measurement points and leg position

**DOI:** 10.17221/8/2024-VETMED

**Published:** 2024-06-27

**Authors:** Tina Bobic, Pero Mijic, Maja Gregic, Vesna Gantner

**Affiliations:** *Department for Animal Production and Biotechnology, Faculty of Agrobiotechnology Sciences Osijek, University of Josip Juraj Strossmayer of Osijek, Osijek, Croatia*

**Keywords:** coronary band, Holstein cows, skin, infrared thermography, lameness

## Abstract

In order to determine the possibility of using infrared thermography (IRT) in preventing lameness in dairy cows, the aim of this study was to evaluate temperature variations depending on the lesion presence, measurement points and leg position. The study used about 3 000 IRT records from 60 Holstein cows housed in a free-stall barn. Surface temperature measurements were taken at two regions of the hooves: the region of the coronary band, and the region of the skin up to 2 cm above the coronary band. A highly significant (*P* = 0.004; 0.006, *P* < 0.01) difference in temperature was observed between healthy and diseased legs at both measurement points. Additionally, a significant (*P* = 0.029; 0.037; 0.045; 0.012; 0.018, *P* < 0.05) or highly significant (*P* = 0.004; 0.006, *P* < 0.01) difference in temperature values between the front and rear legs was established for both measurement points, i.e., the coronary band and the skin. Despite confirming the potential applicability of infrared thermography in the detection of lameness, it is crucial to consider the significant influences of the environmental factors, impurities, and animal-related factors.

Impaired hoof health and lameness in dairy cattle are among the most common problems in milk production, disrupting animal welfare, inducing a drop in milk production (Archer et al. 2010) and finally increasing breeding costs, resulting in significant economic losses (Ozsvari 2017). Hoof problems have a negative impact on the longevity, reproduction and dairy production (Blowey 2012; Solano et al. 2016). According to Garcia-Munoz et al. (2017), the early identification of clinical signs and the timely treatment of hooves is very important for reducing and preventing lameness on the farm. The early detection of infectious conditions, such as digital dermatitis, and effective treatments are crucial to minimising the pain and discomfort associated with lameness (Alsaaod et al. 2014; Pedersen and Wilson 2021). Leach et al. (2012) and Stokes et al. (2012) emphasise how the early detection and treatment of infectious conditions that lead to lameness will not only prevent the progression of the condition, but also reduce the level of infection within the herd. Body extremities and surface temperatures mainly depend on blood circulation and tissue metabolism (Berry et al. 2003). Changes in blood flow affect the amount of radiated heat, especially in the situation when some inflammation is present in the animal’s body. Therefore, changes in these heat amounts can be detected by infrared thermography (Eddy et al. 2001). Infrared thermography (IRT) is a non-invasive diagnostic tool for visualising and determining differences in the surface temperature of the measured body or object (Alsaaod and Buscher 2012) and can be a useful tool in dairy farming management. Previous research with IRT has shown its potential to: detect diseases/disorders of hooves associated with lameness (Nikkhah et al. 2005; Bobic et al. 2018), detect foot-and-mouth disease in cattle (Rainwater-Lovett et al. 2009), detect bovine respiratory disease (Schaefer et al. 2012), identify hoof diseases (Bobic et al. 2017), diagnose dermatitis (Alsaaod et al. 2014) and detect lameness in cattle (Lin et al. 2018).

In order to determine the possibility of using IRT in preventing lameness in dairy cows, the aim of this study was to evaluate the temperature differences depending on the lesion presence, measurement points and leg position.

## MATERIAL AND METHODS

### Data collections

**Figure 1:**
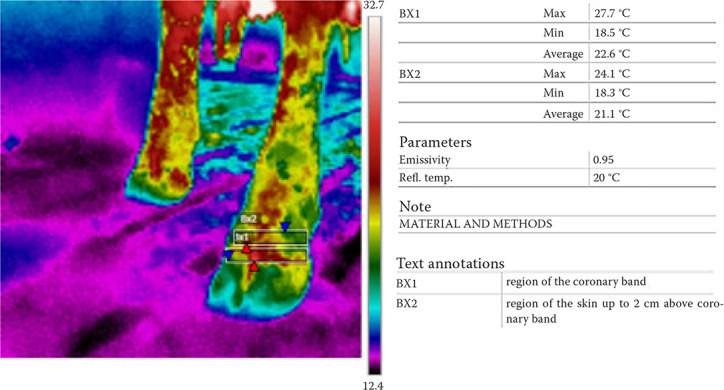
Measurement points marked on the cow’s hooves

The study used about 3 000 IRT records from 60 Holstein cows in their 1^st^ to 6^th^ parity and lactation stages ranging from 10 to 380 days. The research was conducted over three months. The cows were evaluated every seven days. The cows were housed in free-stall barns (cubicle beds). All the examined cows exhibited no visually noticeable changes in the hooves or apparent problems with movement (lameness) (a locomotion score assessment was conducted in the barn before the cows entered the waiting area). The cow’s legs were prewashed with water in a waiting area 30 min before measuring. In the walking corridor before entering the milking facility, the surface temperature of the hooves (the region of the coronary band and the region of the skin up to 2 cm above the coronary band, Figure 1) was measured using a thermovision camera (FLIR i7, FLIR Systems, Inc., Boston, USA) with 0.95 emissivity and an ambient temperature between 19–21 °C. Measurements were taken from a distance of one meter from the front side of the animal’s leg when the cow was standing and waiting to enter the milking area. Throughout the process, one person examined the animals with the thermovision camera. When a suspicious reading (a higher temperature in a certain region compared to other regions) appeared on the camera, the cow was separated for a more detailed examination of the hooves because it was suspected that a tissue change had started. The separated cows were assessed by an official employee trained for the inspection and correction of hooves on the farms (the licensed person for trimming hooves). Cows with confirmed tissue changes on their hooves (such as sole ulcers, interdigital hyperplasia, dermatitis digitalis/interdigitalis) were classified as “diseased”, while those without any confirmed tissue changes were classified as “healthy”. The confirmation of the existence of changes in the hooves and what kind of changes were made by a doctor of veterinary medicine.

### Thermal images data analysis

For the analysis of the thermal images and the determination of the temperature of the hoof’s skin surface, FLIR software (FLIR i7, FLIR Systems Inc., Boston, USA) was used. The temperature measurements of the coronary band and the skin above the coronary band are shown in Figure 1.

### Statistical data analysis

A statistical analysis was conducted using the TIBCO Statistica software package (StatSoft 2020). An analysis of variance (ANOVA) was used to characterise the statistically significant differences between the temperature values of the hoof depending of the presence of tissue changes (lesion and non-lesion), different measurement points (coronary band and skin above the coronary band) and leg position (front and rear, left and right). The significance of the differences between the groups was tested using Fisher’s test at the levels of 0.05 and 0.01.

## RESULTS

Table 1 presents the basic statistical data of the temperature values of the cows’ hooves. The minimum values of the coronary band were 16.0 and 16.1 °C on the front legs, while, on the rear legs, those values were higher, ranging between 21.7 °C to 22.6 °C. The average temperature values of the coronary band were in the range from 24.5 °C to 26.6 °C. The front legs had lower values compared to the rear legs by about 2 °C. The highest maximum temperature of the coronary band was in the rear left legs (34.3 °C), while the lowest was recorded on the front right legs (31.4 °C).

**Table 1:** Descriptive statistics for the temperature of the coronary band and skin

Position of the leg	X	MIN	MAX	SD	CV	SE
	Coronary band					
FL	24.5	16.1	32.8	4.1	16.6	0.7
FR	24.5	16.0	31.4	4.0	16.2	0.7
RL	26.5	21.7	34.3	3.2	11.9	0.6
RR	26.6	22.6	32.1	2.4	9.2	0.4
	Skin					
FL	24.2	15.3	31.5	3.9	16.3	0.7
FR	24.3	14.6	31.2	3.7	15.2	0.7
RL	26.6	21.9	33.7	3.1	11.6	0.6
RR	26.5	21.9	32.1	2.7	10.1	0.5

CV = coefficient of variability; FL = front left leg; FR = front right leg; MAX = maximum value; MIN = minimum value; RL = rear left leg; RR = rear right leg; SD = standard deviation; SE = standard error; X = average value

For the skin temperature (up to 2 cm above the coronary band), the average values ranged from 24.2 °C to 26.6 °C. As was the case with the coronary band, the rear legs also exhibited higher average values by about 2 °C compared to the front legs (Table 1). The minimum temperature values of the skin on the front legs ranged from 14.6 °C to 15.3 °C, while on the rear legs the temperatures were higher, ranging from 21.9 °C. The maximum skin temperature of the rear legs reached values of 32.1 and 33.7 °C, which is higher by about 2 °C compared to the front legs, which recorded temperatures of 31.2 and 31.5 °C.

**Figure 2:**
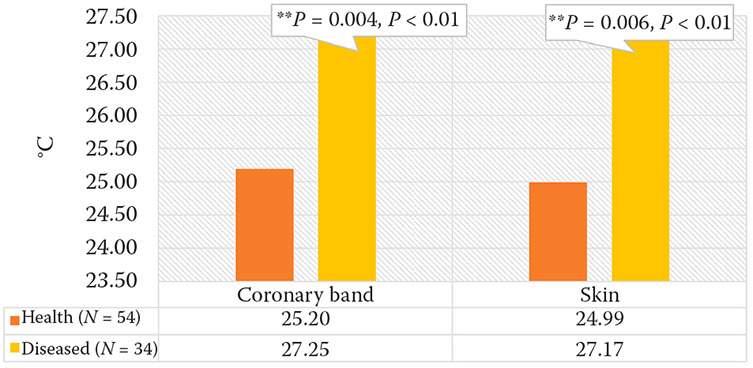
Temperature differences between the healthy and diseased legs depending on the measurement points

The temperature differences between healthy and diseased legs, depending on the measurement points, are presented in Figure 2. After the statistical analyses, a highly significant difference (*P* < 0.01) in the temperature for both measurement points between the healthy and diseased legs was observed. The temperature of the coronary band of the cows’ legs which had some lesions (diseased legs) had highly significant higher values (*P* = 0.004, *P* < 0.01) compared to those of the healthy cows’ legs (27.25 : 25.20).

Furthermore, the temperature of the skin on the healthy legs was highly significantly lower (*P* = 0.006, *P* < 0.01) than the skin temperature of the diseased legs (24.99 : 27.17).

In this research, the influence of the leg position on the values of the hoof temperature was also analysed. Significantly (*P* = 0.029; 0.037; 0.045, *P* < 0.05) or highly significantly (*P* = 0.004; 0.006, *P* < 0.01) different temperature values were established between the front and rear legs for both measurement points, the coronary band and skin (Table 2). It was concluded that the front legs had significantly lower temperature values of the coronary band and skin above the coronary band compared to those measurement points on the rear legs. For the temperature of the coronary band, a significant difference (*P* = 0.029; 0.037, *P* < 0.05) of about 3 °C was found between the left front and both rear left and right legs (24.84 : 27.10; 24.84 : 26.99 °C), respectively. While the front right legs had significantly (*P* = 0.045, *P* < 0.05) lower values by about 2 °C compared to rear left legs (25.03 : 27.10 °C), there was no significant difference compared to the rear right legs (25.03 : 26.99 °C). The skin temperature of the front left legs was highly significantly lower (*P* = 0.004; 0.006, *P* < 0.01) compared to the skin of both rear legs, with a difference of about 3 °C (24.34 : 27.21; 24.34 : 27.06 °C). As for the front right legs, significant differences were found (*P* = 0.012; 0.018, *P* < 0.05) compared to both rear legs (24.74 : 27:21; 24.74 : 27.06 °C, Table 2). No significant temperature difference was found between the hooves of the front legs for both measurement points (*P* = 0.848; 0.680, *P* > 0.05; *P* > 0.01).

**Table 2:** Temperature differences between the measurement points according to the leg position (ANOVA)

Trait	Leg position				*P*-values
	front		rear		
	left	right	left	right	
Coronary band	24.84^a*^	25.03^a,c*^	27.10^b*^	26.99^b,c*^	*P* = 0.029^*^; 0.037^*^; 0.045^*^
Skin	24.34^a**^	24.74^a*:**^	27.21^b*;**^	27.06^b*:**^	*P*** = **0.004^**^; 0.006^**^; 0.012^*^; 0.018^*^

*Values within the same row marked with different letters differ statistically significantly (*P* < 0.05); **Values within the same row marked with different letters differ statistically highly significantly (*P* < 0.01)

## DISCUSSION

Detecting the inflammatory process in cows’ hooves is of great importance, especially during early lactation, because it affects the milk production (Racewicz et al. 2018). According to Poikalainen et al. (2012) and Werema et al. (2023), the temperature of specific body parts can be used as a good indicator in assessing the health and physiological status of cows. Alsaod et al. (2015) also emphasised that variations in superficial thermal values resulting from changes in blood flow can be used to detect inflammation or injury associated with conditions such as foot lesions. Thermography can be used to identify and determine these thermal abnormalities in animals by characterising increases or decreases in the surface temperature of their skin. The results of this study confirm the possibility of using IRT in detecting cows with thermal abnormalities and foot lesions, as significant temperature differences were found between the healthy and diseased legs. The temperature of the coronary band and skin of cows’ diseased legs had highly significant (*P* = 0.004; 0.006*, P* < 0.01) higher values compared to those of the healthy cows’ legs, which is consistent with the findings of Alsaaod and Buscher (2012). The diseased legs had elevated temperatures of more than 2 °C compared to the healthy legs, regardless of the measurement points. For the coronary band, this was 27.25 : 25.20 °C and 24.99 : 27.17 °C for the skin temperature. These results were similar to those of Bobic et al. (2017), who also reported significantly higher average temperatures of the coronary band in the hooves with lesions compared to the healthy ones (18.62 : 22.76 °C), but their differences were higher (>3 °C : >2 °C) compared to the findings on this research (18.62 : 22.76 °C : 27.25 : 25.20 °C). All of the above is consistent with the previous results of the researchers Schaefer and Cook (2013) and Alsaod et al. (2014) on the potential use of IRT in detecting lameness. The reliability of using IRT has been confirmed in previous research, such as that of Wilhelm et al. (2015), who determined a threshold value of the 25.25 °C (plantar view), with 72% sensitivity and 73% specificity, while Alsaaod and Buscher (2012) had 85.7 and 80.0% sensitivity for the coronary band (dorsal view) and 55.9 and 82.9% specificity for the skin (dorsal view). Furthermore, Alsaaod et al. (2014) established a sensitivity value for the coronary band and skin (lateral and medial view) of 89.1% and a specificity of 66.6%.

In this research, different values of detected leg temperature were observed regarding the leg position, which means that the front legs had a lower temperature by approximately 3 °C in comparison with the rear legs. This aligns with the research of Nikkah et al. (2005), who suggested that dairy cows have an uneven body mass distribution, with the rear part of the body being heavier, and that is the reason why the rear legs should be stronger and more vascularised. Accordingly, it is assumed that they have increased circulation, which can be associated with the higher temperatures observed in the rear legs compared to the front legs. In addition to the leg position, Werema et al. (2023) determined the effect of the different parts of the hooves on the surface temperature; they detected a higher mean temperature for the zones on the lateral hoof compared to their equivalents on the medial hoof.

Despite the confirmed potential applicability of IRT in detecting lameness, it is crucial to consider the significant influences of environmental factors, impurities, and animal-related factors. For example, Lokesh Babu et al. (2018) emphasised that the appropriate preparation of the animals and environmental conditions must be confirmed before thermographic examination, e.g., the area must be free from direct sunlight and draughts, the feet should be dry and clean, the camera must be held at the same angle and distance from the target point, etc.

Furthermore, Gloster et al. (2011) determined that the ambient temperature and the animal’s activity immediately prior to measurement primarily influence the animal’s hoof temperature.

By analysing the presented results, it can be concluded that it is possible to detect temperature differences in the tissue of the cows’ hooves with an infrared thermovision camera. The temperature values of the cows’ hoof tissue was influenced by the presence of lesions and the position of the leg.

The early identification of clinical signs and the timely treatment of hooves is very important in reducing and preventing lameness on the farm. Improving cattle comfort, welfare and reducing economic costs are essential for successful dairy farming. This can be achieved by using new technologies such as infrared thermography. Despite the confirmed potential applicability of the infrared thermography in detecting lameness, it is crucial to consider the significant influences of environmental factors, impurities, and animal-related factors.
